# Pediatric Non-cystic Fibrosis Bronchiectasis in a Portuguese Tertiary Care Center: A Cross-Sectional Observational Study

**DOI:** 10.7759/cureus.78551

**Published:** 2025-02-05

**Authors:** Sara Nogueira Machado, Sofia Branco, Sónia Silva, Catarina Ferraz, Inês Azevedo

**Affiliations:** 1 Department of Pediatrics, Unidade Local de Saúde do Alto Ave, Guimarães, PRT; 2 Department of Pediatrics, Unidade Local de Saúde Póvoa de Varzim/Vila do Conde, Póvoa de Varzim, PRT; 3 Department of Pediatrics, Hospital da Povoa, Póvoa de Varzim, PRT; 4 Pediatric Pulmonology Unit, Department of Pediatrics, Unidade Local de Saúde de São João, Porto, PRT; 5 Department of Gynecology-Obstetrics and Pediatrics, Faculdade de Medicina, Universidade do Porto, Porto, PRT

**Keywords:** chronic wet cough, etiology, non-cystic fibrosis bronchiectasis, pediatric bronchiectasis, primary ciliary dyskinesia

## Abstract

Introduction

Non-cystic fibrosis bronchiectasis (bronchiectasis) is an increasingly recognized but understudied disease in children. National data on this disease are scarce. This study aimed to describe the clinical, radiological, and microbiological characteristics of Portuguese children with bronchiectasis.

Methods

A retrospective observational study was conducted at a tertiary pediatric pulmonology center in northern Portugal. Pediatric patients diagnosed with bronchiectasis and followed between July 2020 and September 2023 were included.

Results

A total of 38 patients were included, of whom 19 (50.0%) were male, with a median age at diagnosis of 6.3 years (3.8-11.0 years). Recurrent wheezing (n = 30, 78.9%) and chronic wet cough (n = 18, 47.4%) were the most common symptoms. An underlying etiology was identified in 36 (94.7%) patients, primarily postinfectious bronchiectasis (n = 18, 47.4%) and primary ciliary dyskinesia (n = 10, 26.3%). Multilobar involvement was observed in 25 (65.8%) patients, most frequently affecting the middle and lower lobes. Spirometry showed a mixed obstructive-restrictive pattern in 10 (33.3%) patients and a predominantly obstructive pattern in nine (30.0%) patients. *Haemophilus influenzae* and *Streptococcus pneumoniae* were the most frequently isolated microorganisms, both in bronchoalveolar lavage and sputum cultures. *Pseudomonas aeruginosa* was detected in nine (7.4%) sputum samples.

Conclusion

This study highlights the diverse clinical presentations, etiologies, and microbiological findings in pediatric bronchiectasis. Identifiable causes were present in most cases, emphasizing the importance of clinical vigilance for early diagnosis and intervention. Further research is warranted to explore long-term outcomes and refine treatment approaches based on microbiological profiles.

## Introduction

Bronchiectasis is a chronic pulmonary syndrome characterized by abnormal, progressive, and often irreversible bronchial dilatation on chest computed tomography (CT) scans [1,2]. Although traditionally associated with cystic fibrosis (CF), bronchiectasis unrelated to CF is increasingly recognized as a significant cause of chronic respiratory morbidity in children in both developed and developing countries [3]. The incidence of pediatric bronchiectasis is not well-defined, with recent estimates ranging from 0.2 to 735 per 100,000 children per year worldwide, reflecting differences in healthcare access, diagnostic practices, and underlying etiologies [4].

Bronchiectasis is often the end result of multiple conditions that lead to recurrent or persistent lower respiratory tract infections [5]. Most pediatric bronchiectasis cases have an identifiable etiology, with infections, primary ciliary dyskinesia (PCD), immunodeficiencies (primary or acquired), and aspiration (both foreign body and recurrent aspiration in neurodevelopmentally challenged patients) being the most frequent ones [6,7]. The clinical presentation of bronchiectasis in children is highly variable, often resulting in delays in diagnosis and treatment. Chronic wet cough is the most common symptom and it may persist for months or years before the diagnosis is established or even considered [4]. Other symptoms, such as recurrent wheezing, recurrent lower respiratory tract infections, persistent purulent sputum production, and, less commonly, hemoptysis, digital clubbing, or chest wall deformity, may also be present, further increasing the likelihood of underlying bronchiectasis [1,4].

A high-resolution chest CT scan is the gold-standard imaging method for the diagnosis of bronchiectasis, as it provides the most reliable assessment of bronchial dilatation [8]. Chronic bacterial colonization is frequently observed in children with bronchiectasis, with *Haemophilus influenzae*, *Streptococcus pneumoniae*, and *Moraxella catarrhalis* being the most commonly isolated microorganisms. In contrast with CF, *Staphylococcus aureus* and *Pseudomonas aeruginosa* are less frequently detected in these patients, and their presence often reflects more advanced disease [5,9].

Current bronchiectasis treatment strategies are primarily supportive, focusing on preventing the cycle of infection and inflammation, and consequently reducing the frequency of pulmonary exacerbations and improving lung function [4]. Despite the dearth of evidence on treatment strategies, airway clearance techniques, along with systemic or inhaled antibiotics, are often considered the cornerstones of pediatric bronchiectasis management and are routinely recommended for children with this condition [2,4].

Despite its growing recognition, in Portugal, pediatric bronchiectasis remains an understudied and often neglected disease, and national data on this condition are scarce. We aimed to address this gap by describing the clinical, radiological, and microbiological characteristics of a cohort of children with bronchiectasis followed at a tertiary pediatric center in northern Portugal.

This study was previously presented as an oral communication at the 2^nd^ Congress of the International Society of Pediatric Respiratory Diseases, on July 6, 2024.

## Materials and methods

We conducted a single-center, retrospective, observational cohort study. All patients aged 18 years or less with a diagnosis of bronchiectasis confirmed by chest CT scan and followed at the outpatient department of a tertiary pediatric pulmonology center in northern Portugal between July 1, 2020, and September 30, 2023, were included. Exclusion criteria comprised a concurrent diagnosis of CF or loss of follow-up.

Electronic clinical records of the included patients were reviewed to collect data on sex, age at diagnosis, duration of follow-up, clinical signs and symptoms, physical examination findings, etiology, type and localization of bronchiectasis, pulmonary function tests, microbiological evaluations, and medical treatments. Data collection was cross-checked by two independent reviewers to ensure accuracy and consistency. Age at diagnosis was defined as the date of the first radiologic confirmation of bronchiectasis.

CF was excluded by a normal sweat test or a negative genetic analysis [10]. The diagnosis of primary ciliary dyskinesia was confirmed through unequivocally abnormal findings on transmission electron microscopy or biallelic disease-causing mutations identified through genetic testing [11]. Immunocompetence was assessed by a basic immunological panel (full blood count, IgG, IgA, IgM, IgE, and antibodies subclasses) in all patients. T lymphocyte subsets and neutrophil function and specific antibodies to vaccine antigens were performed in selected cases. Bronchiectasis was classified as postinfectious if there was a history of a severe or recurrent respiratory infection, after ruling out other potential causes of bronchiectasis, or as idiopathic when no underlying etiology was identified.

Bronchiectasis diagnosis was established by chest CT scans and defined by the presence of bronchial dilatation (internal bronchial diameter exceeding the adjacent pulmonary artery) and a lack of bronchial tapering on sequential slices [5]. The morphological types of bronchiectasis were classified according to Reid’s criteria [12].

All collaborating patients performed sputum cultures routinely and during exacerbations, whenever possible. Bacterial colonization was defined as the detection of the same pathogen in sputum samples on at least two occasions, at least three months apart, within one year [13].

Follow-up of bronchiectasis in our center includes visits every three to six months in stable patients, with additional visits during exacerbations if possible. Exacerbations were defined by an increase in symptoms such as cough and sputum production, or the emergence of more severe symptoms, including hemoptysis, chest pain, or dyspnea.

Continuous variables were not normally distributed and are presented as medians and interquartile ranges (IQRs), represented as the 25^th^ (Q1) and 75^th^ (Q3) percentiles. Categorical variables are presented as frequencies and percentages. Statistical analysis was performed using the IBM SPSS Statistics software version 24.0 (IBM Corp., Armonk, NY).

This study was conducted in accordance with all relevant national regulations and institutional policies, and it complies with the tenets of the Helsinki Declaration. Informed consent for this study was waived by the local Institutional Review Board as data were anonymized and collected retrospectively as part of routine care. Data access was approved by the institution's Information Access Officer on November 14, 2024, under approval number 24019543.

## Results

A total of 38 pediatric patients were included in the study. Half of the patients (n = 19, 50.0%) were male, with a median age of 6.3 (3.8-11.0) years at diagnosis. Thirty patients (78.9%) had a history of recurrent wheezing or asthma, and 17 (44.7%) had experienced recurrent lower respiratory tract infections. Chronic wet cough and persistent purulent sputum production were commonly reported symptoms, occurring in 18 (47.4%) and 14 (36.8%) patients of the cohort, respectively. None of the patients presented with hemoptysis, digital clubbing, or chest wall deformity. A comprehensive detailed summary of the demographic and clinical characteristics of the study participants is provided in Table 1.

**Table 1:** Demographic and clinical characteristics of the patients. ^a ^Median (Q1-Q3).

Variables	Frequencies
Male sex, n (%)	19 (50.0)
Age at diagnosis (years)^ª^	6.3 (3.8-11.0)
Duration of follow-up (years)^ª^	7.3 (4.2-10.7)
Clinical manifestations, n (%)	
Recurrent wheezing/asthma	30 (78.9)
Chronic wet cough	18 (47.4)
Recurrent lower respiratory tract infections	17 (44.7)
Persistent purulent sputum production	14 (36.8)
Dyspnea	5 (13.2)
Sinusitis	4 (10.5)
Hemoptysis	0 (0.0)
Physical examination findings, n (%)	
Chest wall deformity	0 (0.0)
Digital clubbing	0 (0.0)

Postinfectious bronchiectasis (n = 18, 47.4%) and PCD (n = 10, 26.3%) accounted for the majority of the cases in this cohort. Six out of the 18 postinfectious cases (15.8%) had other typical findings consistent with bronchiolitis obliterans on chest CT scans, with four of these being secondary to adenovirus infection. Chronic aspiration syndrome, associated with feeding or swallowing difficulties due to tracheoesophageal fistula or cerebral palsy, was identified in four cases (10.5%). Table 2 further details the underlying causes of bronchiectasis.

**Table 2:** Underlying etiology of bronchiectasis. ^†^ Common variable immunodeficiency, hyper-immunoglobulin M syndrome, cardiofaciocutaneous syndrome. ^‡^ Carbamazepine-induced interstitial pneumonitis associated with pan-hypogammaglobulinemia.

Variables	Frequencies
Postinfectious, n (%)	18 (47.4)
Postinfectious bronchiolitis obliterans	6 (15.8)
Other postinfectious causes	12 (31.6)
Primary ciliary dyskinesia, n (%)	10 (26.3)
Chronic aspiration syndrome, n (%)	4 (10.5)
Tracheoesophageal fistula	3 (7.9)
Cerebral palsy	1 (2.6)
Primary immunodeficiency^†^, n (%)	3 (7.9)
Drug-induced^‡^, n (%)	1 (2.6)
Idiopathic, n (%)	2 (5.2)

**Figure 1:**
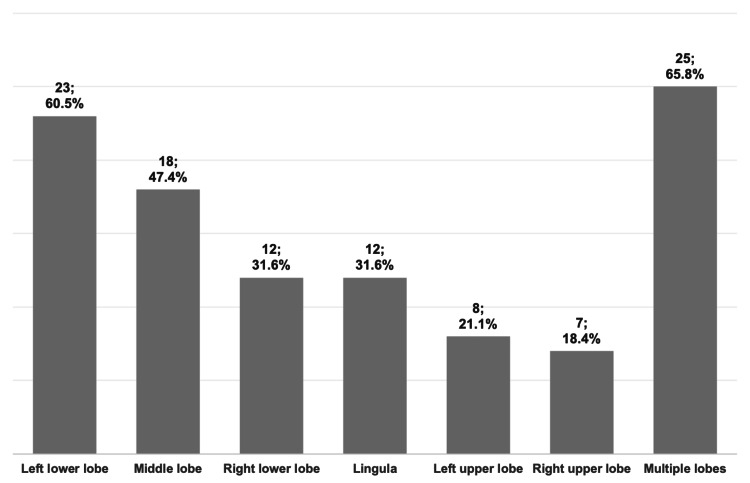
Localization of bronchiectasis on chest CT scans.

**Figure 2:**
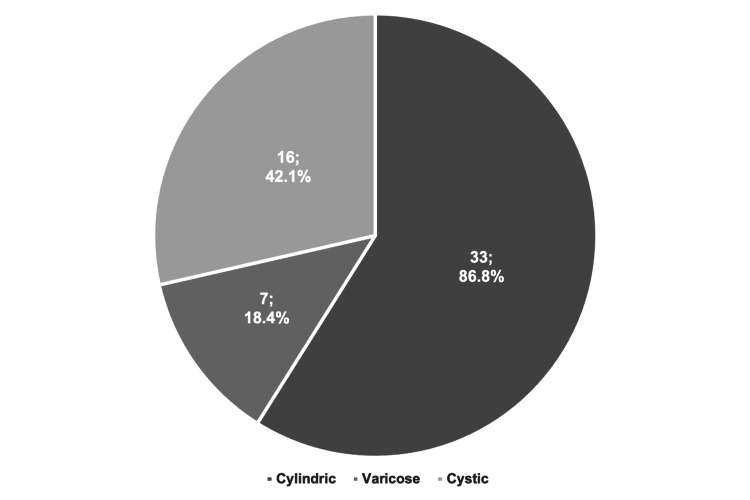
Type of bronchiectasis.

The lower left lobe was the most frequently affected (n = 23, 60.5%), and multilobar involvement was observed in 25 patients (65.8%), as shown in Figure 1. Regarding the type (Figure 2), cylindric bronchiectasis was the most common, seen in 33 patients (86.8%).

After diagnosis, 30 (78.9%) patients underwent at least one pulmonary function test. Spirometry results revealed a mixed ventilatory syndrome in 10 (33.3%) patients, an obstructive pattern in nine (30.0%), and a restrictive pattern in four (13.3%) patients. The remaining patients (n = 7, 23.3%) had normal spirometry results.

Of the 17 (44.7%) patients who underwent a bronchoscopy, airway suppuration was observed in 12 (70.6%) in variable degrees. *Haemophilus influenzae* was identified in 10 of these cases (83.3%). During follow-up, 28 patients provided 121 valid sputum samples: *Haemophilus influenzae* (46 samples, 38.0%), *Streptococcus pneumoniae *(16 samples, 13.2%), methicillin-susceptible *Staphylococcus aureus* (10 samples, 8.3%), and *Pseudomonas aeruginosa* (nine samples, 7.4%) were the most frequently identified pathogens. Four patients were classified as chronically colonized with the following microorganisms: *Haemophilus influenzae* (n = 2), *Pseudomonas aeruginosa *(n = 2), methicillin-susceptible *Staphylococcus aureus *(n = 1), and *Streptococcus pneumoniae* (n = 1). Further details are available in Table 3.

**Table 3:** Lower airway microbiological findings. ª Median (Q1-Q3). ^†^ *Haemophilus parainfluenzae*, *Stenotrophomonas maltophilia*, *Acinetobacter junii*, *Streptococcus pyogenes*, and *Citrobacter freundii*.

Variables	Frequencies
Bronchoscopy, n (%)	
Performed	17 (44.7)
Normal	5 (29.4)
Airway suppuration	12 (70.6)
Not performed	21 (55.3)
Microorganisms identified, n (%)	
Haemophilus influenzae	10 (83.3)
Streptococcus pneumoniae	2 (16.7)
Methicillin-susceptible Staphylococcus aureus	1 (8.3)
Morganella morganii	1 (8.3)
Pseudomonas aeruginosa	1 (8.3)
Sputum cultures	
Number of samples collected, n	121
Number of samples per patient^ª^	1.5 (0.0-4.0)
Microorganisms identified, n (%)	
Haemophilus influenzae	46 (38.0)
Streptococcus pneumoniae	16 (13.2)
Methicillin-susceptible Staphylococcus aureus	10 (8.3)
Pseudomonas aeruginosa	9 (7.4)
Moraxella catarrhalis	3 (2.5)
Other pathogens^†^	5 (4.1)

Regarding the treatment, 10 (26.3%) patients were being treated with azithromycin, primarily during the winter months. One (2.6%) patient with hyper-immunoglobulin M syndrome was under long-term prophylaxis with trimethoprim/sulfamethoxazole. Twenty (52.6%) subjects were prescribed inhaled corticosteroids, with 14 (36.8%) also using a long-acting beta-agonist. Sixteen (42.1%) patients regularly used mucoactive agents, specifically hypertonic saline, and were reported to receive regular airway clearance techniques. No patients underwent surgical treatment for their bronchiectasis.

## Discussion

This retrospective study provides a comprehensive overview of the clinical, radiological, and microbiological characteristics of a group of 38 children with bronchiectasis followed at a tertiary pediatric center in northern Portugal.

The median age at diagnosis was 6.3 years, which is consistent with similar studies from both developed and developing countries. The wide range in age at diagnosis also aligns with existing literature and underscores the variable onset and progression of pediatric bronchiectasis, which can pose challenges for early detection and timely intervention [1,8,14-17]. There were no differences in sex distribution, which is consistent with most studies [14,17-20], although a study in Sri Lanka [16] found a higher prevalence in females and some studies [1,21] reported a slight prevalence in males.

Compared to previous studies [1,5,7,15,17,21,22], our cohort exhibited a notably lower incidence of chronic wet cough and persistent purulent sputum production (47.4% and 36.8%, respectively), the hallmark symptoms of bronchiectasis. As this study relied solely on the review of clinical records, these findings are likely attributed to under-reporting or under-registration of symptoms, a limitation often encountered in retrospective studies. A high incidence of recurrent wheezing or asthma (78.9%) was observed, probably due to the not-so-infrequent overlap between bronchiectasis and other common pediatric respiratory conditions, such as asthma [4,8,22]. The pathophysiological mechanisms by which asthma and bronchiectasis are associated remain unclear, but a previous poorly controlled asthma could ultimately increase the risk of respiratory complications, such as bronchiectasis [23]. Our results reinforce the heterogeneous clinical presentation of pediatric bronchiectasis and highlight the importance of maintaining a high index of suspicion when evaluating children with persistent respiratory symptoms.

Notably, none of the patients presented with hemoptysis, digital clubbing, or chest wall deformity. The absence of these advanced symptoms suggests that bronchiectasis may have been diagnosed at an earlier stage, before progressing toward more severe manifestations. Further research is needed to assess whether this reflects improved clinical awareness or differences in disease severity at presentation.

The diverse etiological spectrum of bronchiectasis observed in our cohort reflects the multifactorial nature of the condition. As similarly reported in other studies [5,6,14,20,22], most cases in this sample had an identifiable cause, with postinfectious bronchiectasis and PCD being the most prevalent (47.4% and 26.3%, respectively). While the widespread use of antibiotics and early childhood vaccination has led to a decline in the incidence of infectious diseases, particularly in developed countries like Portugal, the significant proportion of cases resulting from severe or recurrent lower respiratory tract infections in this cohort highlights the ongoing importance of infections as a critical etiological factor [8]. This finding emphasizes the importance of prompt and effective management of respiratory infections in children to prevent progression to bronchiectasis. The prominent role of PCD in this series also aligns with recent studies reporting a rising prevalence of this condition in developed countries. The increased availability of advanced diagnostic testing such as high-speed videomicroscopy, electron microscopy, and genetic analysis has enabled an accurate and earlier diagnosis of PCD [14,19,24]. The diagnosis of aspiration syndrome, identified in four cases as the underlying etiology of bronchiectasis, underscores the need for careful monitoring of children at risk of recurrent aspiration. Similarly, the diagnosis of an immunodeficiency, although less common in this series, highlights the importance of thorough immunological evaluations in children with unexplained bronchiectasis. Notably, the prevalence of idiopathic bronchiectasis was lower than expected [5,14], given that an underlying etiology failed to be identified in only two cases (5.2%).

The radiological findings in the present study align with existing literature on pediatric bronchiectasis. In this cohort, the middle and lower lobes were frequently involved, whereas the upper lobes were relatively spared. This observation is in accordance with multiple studies reporting that bronchiectatic lesions unrelated to CF are most frequently found in the lower lobes, probably because mucociliary clearance is facilitated by gravity in the upper lobes [8,15,17,20]. Consistent with reports from Saudi Arabia, Turkey, Italy, and New Zealand [17-19,25], multilobar involvement was predominant in this cohort (65.8% of cases). Regarding the phenotype, cylindrical bronchiectasis was the most frequently observed (86.8%), confirming its status as the major form of bronchiectasis in pediatric cases [26]. However, a significant proportion of patients presented with varicose (18.4%) and/or cystic bronchiectasis (42.1%), possibly indicating a more advanced and severe condition.

Pulmonary function tests are one way of defining lung disease severity and progression and should be routinely performed in all collaborating children with bronchiectasis [2,4]. In this cohort, most patients (78.9%) underwent at least one spirometry after the diagnosis, revealing an abnormal lung function in a significant proportion, with a predominance of mixed obstructive and restrictive (33.3%), as well as primarily obstructive (30.0%) patterns. While a predominantly obstructive lung disease has been described in other pediatric bronchiectasis case series [4,27,28], the higher prevalence of mixed obstructive and restrictive patterns in this cohort likely indicates more advanced stages of lung disease. Interestingly, the lung function trajectory in pediatric bronchiectasis varies across studies. While Twiss et al. [27] reported that children with bronchiectasis had significant airway obstruction that deteriorates over time, McCallum et al. [29] and Bastardo et al. [28] found that most children receiving specialized care had stable lung function within the normal range. These differences are likely attributable to differences in underlying etiology, disease severity, ethnicity, and management strategies [30]. This variability underscores the complexity of bronchiectasis and highlights the need for further research to better understand lung function trajectories in children with bronchiectasis.

Bacterial colonization and infection of the lower airways contribute to the cascade of events that culminate in bronchial damage with dilatation [20]. For that reason, the European Respiratory Society pediatric bronchiectasis guidelines recommend routine microbiological assessments for identifying new pathogens and guiding empiric antibiotic therapy in future exacerbations [2]. In this cohort, *Haemophilus influenzae* and *Streptococcus pneumoniae* were the most frequently isolated microorganisms, both in bronchoalveolar lavages and sputum cultures. This finding is consistent with recent studies showing that *Haemophilus influenzae* and *Streptococcus pneumoniae* are the major pathogens among pediatric bronchiectasis patients [9,14]. The significant prevalence of other pathogens, including *Moraxella catarrhalis* and methicillin-sensitive *Staphylococcus aureus*, also aligns with other studies that emphasize the polymicrobial nature of pediatric bronchiectasis [4,9,14,31]. Of particular concern is the detection of *Pseudomonas aeruginosa* in one (8.3%) bronchoalveolar lavage and nine (7.4%) sputum samples. *Pseudomonas aeruginosa* is an opportunistic pathogen whose presence in the lower airways is associated with an increased frequency of exacerbations, lower lung function, and more advanced disease [9,30]. These findings underscore the importance of regular microbiological surveillance and tailored antibiotic therapy to effectively manage chronic infection and prevent disease progression.

Despite the lack of strong evidence, treatment with antibiotics and airway clearance techniques are usually considered the cornerstones of pediatric bronchiectasis management [2,4]. In this cohort, about one-quarter of the patients (26.3%) were being treated with long-term azithromycin, primarily during the winter months. This approach aligns with current practices in managing pediatric bronchiectasis, where macrolides like azithromycin are often used for their anti-inflammatory and antimicrobial properties, as well as their ability to halve exacerbation frequency [4]. The use of trimethoprim/sulfamethoxazole, although less common, is consistent with its role in preventing *Pneumocystis jirovecii* pneumonia in patients with hyper-immunoglobulin M syndrome [32]. Additionally, 20 patients (52.6%) were on inhaled corticosteroids, with 14 (36.8%) also using a long-acting beta-agonist. The use of inhaled corticosteroids in bronchiectasis, either alone or in combination with long-acting beta-agonists, is controversial since studies show no significant reduction in exacerbation frequency or antibiotic use [2,4]. The high proportion of patients using inhaled corticosteroids in this cohort likely reflects the overlap of bronchiectasis with asthma and other obstructive airway diseases. Sixteen patients (42.1%) used mucoactive agents, specifically hypertonic saline, alongside regular airway clearance techniques. Although not routinely recommended, hypertonic saline is supported by literature indicating its effectiveness in patients with high daily symptoms, frequent exacerbations, and/or difficulty in expectoration [2]. Despite the theoretical importance of airway clearance techniques in bronchiectasis management, such as improving exercise capacity, reducing cough and sputum volumes, and preventing infections [2,4], the application of these techniques was less widespread than desirable in our cohort. Our results align with those of Spinou et al. [33] and are likely explained by the difficulty of access to trained technicians in the patients’ residential areas. This highlights a critical gap in the accessibility of essential therapies that could significantly improve patient outcomes.

This study is not without limitations. First, its retrospective nature may have compromised the recollection of clinical data. Second, the small sample size, while comparable to similar studies, limits the generalizability of the findings and the ability to perform detailed subgroup analyses. Additionally, the single-center design provides limited information on pediatric bronchiectasis across Portugal. Lastly, the limited number of sputum samples per patient, due to a significant number of non-collaborating subjects and the challenges posed by the COVID-19 pandemic, further constrain the generalizability of the results and the potential for in-depth microbiological analysis. Future research should focus on multicenter longitudinal studies to explore lung function trajectories, pathogen-related evolution, and treatment adherence patterns over time. Furthermore, assessing the psychosocial impact of pediatric bronchiectasis, including its effects on school attendance, caregiver stress, and overall quality of life, could provide a more holistic understanding of the disease burden.

## Conclusions

This study provides a detailed characterization of pediatric bronchiectasis in a retrospective cohort from northern Portugal, highlighting the diverse clinical presentations, etiological factors, microbiological findings, and treatment strategies associated with this condition. Future research should explore the national panorama of pediatric bronchiectasis and assess the long-term outcomes linked to different microbiological profiles and treatment approaches, potentially informing more tailored care strategies.
